# Roles of curli, cellulose and BapA in *Salmonella *biofilm morphology studied by atomic force microscopy

**DOI:** 10.1186/1471-2180-7-70

**Published:** 2007-07-24

**Authors:** Kristina Jonas, Henrik Tomenius, Abdul Kader, Staffan Normark, Ute Römling, Lyubov M Belova, Öjar Melefors

**Affiliations:** 1 Swedish Institute for Infectious Disease Control, SE-17182 Solna, Sweden; 2 Department of Microbiology, Tumor and Cell Biology, Karolinska Institutet, SE-17177 Stockholm, Sweden; 3 Department of Cell and Molecular Biology, Uppsala University, SE-751 24 Uppsala, Sweden; 4 Department of Materials Science and Engineering, Royal Institute of Technology, SE-10044 Stockholm, Sweden

## Abstract

**Background:**

Curli, cellulose and the cell surface protein BapA are matrix components in *Salmonella *biofilms. In this study we have investigated the roles of these components for the morphology of bacteria grown as colonies on agar plates and within a biofilm on submerged mica surfaces by applying atomic force microscopy (AFM) and light microscopy.

**Results:**

AFM imaging was performed on colonies of *Salmonella *Typhimurium grown on agar plates for 24 h and on biofilms grown for 4, 8, 16 or 24 h on mica slides submerged in standing cultures. Our data show that in the wild type curli were visible as extracellular material on and between the cells and as fimbrial structures at the edges of biofilms grown for 16 h and 24 h. In contrast to the wild type, which formed a three-dimensional biofilm within 24 h, a curli mutant and a strain mutated in the global regulator CsgD were severely impaired in biofilm formation. A mutant in cellulose production retained some capability to form cell aggregates, but not a confluent biofilm. Extracellular matrix was observed in this mutant to almost the same extent as in the wild type. Overexpression of CsgD led to a much thicker and a more rapidly growing biofilm. Disruption of BapA altered neither colony and biofilm morphology nor the ability to form a biofilm within 24 h on the submerged surfaces. Besides curli, the expression of flagella and pili as well as changes in cell shape and cell size could be monitored in the growing biofilms.

**Conclusion:**

Our work demonstrates that atomic force microscopy can efficiently be used as a tool to monitor the morphology of bacteria grown as colonies on agar plates or within biofilms formed in a liquid at high resolution.

## Background

Many microbes tend to form sessile communities on surfaces, where the cells are embedded in a self-produced matrix of extracellular polymeric substances [1]. These biofilms provoke major concern in many industrial processes and in medicine [1,2]. The formation of biofilms has been described as a developmental differentiation process, which is typically initiated by the attachment of free living bacteria to an exposed surface [2]. Subsequent development often occurs along with the production of a tight network of extracellular polymers that facilitates dense adherence to each other and to the surface leading to a differentiated three-dimensional biofilm architecture [2-5].

**Figure 1:**
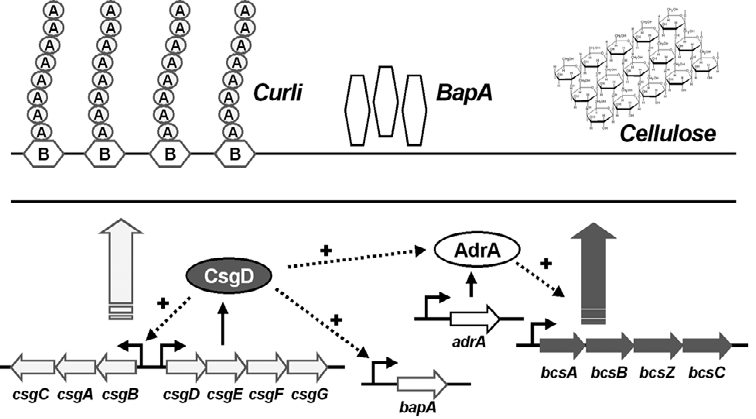
Current model of the regulatory network controlling the expression of extracellular matrix components required for biofilm formation in *Salmonella*. The curli subunits CsgA and CsgB are encoded by *csgBA*, which is positively regulated by the global regulator CsgD [20]. CsgD also activates cellulose production through the expression of AdrA, a member of the GGDEF protein family, which is involved in the activation of cellulose synthesis [15]. Recently, in *S*. Enterica serovar Entiritidis a large secreted protein, called BapA, has been discovered. BapA was shown to be required for pellicle formation at the air-liquid interface and its expression was demonstrated to be coordinated with that of genes encoding curli and cellulose via CsgD [21].

The extracellular matrix can consist of various mixes of polysaccharides, proteins and even nucleic acids [6], where the composition depends on the nature of the contributing species as well as environmental conditions [7]. The food-borne pathogen *Salmonella enterica*, as well as other members from the *Enterobacteriaceae *family, forms biofilms on biotic and abiotic surfaces during their natural life cycles [8,9]. *Salmonella *produces an extracellular matrix with curli as the major proteinaceous component [10]. Curli are amyloid fibers, which are involved among other things in adhesion to surface, cell aggregation, environmental persistence and biofilm formation [11-18]. As a second matrix-component *Salmonella enterica *and pathogenic and commensal *Escherichia coli *strains secrete cellulose [15,19]. Both curli and cellulose synthesis are coregulated by a complex regulatory network (Fig. 1), in which the LuxR type regulator CsgD (also described as AgfD) plays a key role [20]. CsgD stimulates the production of curli through the transcriptional activation of the *csgBAC *operon. The activation of cellulose production is indirect through the regulator AdrA [15,20]. Recently, the discovery of BapA (biofilm-associated protein), a large cell-surface protein required for biofilm formation by *Salmonella enterica *serovar Enteritidis was reported [21]. The expression of *bapA *was demonstrated to be coordinated with the expression of curli and cellulose, through the action of CsgD [21]. CsgD has also been demonstrated to regulate expression of the genes for an O-antigen capsule [22]. This capsule was not important for multicellular aggregation, but for environmental persistence [22].

Bacteria expressing curli and cellulose display a red, dry and rough colony morphology (rdar) on Congo red agar plates. Disruption of one or both of these components leads to the development of distinct colony morphology types [23]. Deficiency in curli and cellulose synthesis causes a smooth and white (saw) colony appearance, a defect in cellulose synthesis curli leads to brown (bdar) colonies and a defect in curli expression to a pink (pdar) morphotype [23].

Besides Congo red plate assays, other methods, such as electron microscopy, liquid-air pellicles and microtiterplate biofilm assays have been used to characterize the multicellular behaviour of *Salmonella *and to demonstrate that curli, cellulose and BapA are important for biofilm formation. However, in these assays the distinct roles of the extracellular matrix components in time-dependent formation of the biofilm morphology have not been studied. In this study we have in detail characterized the impact of mutations in curli, cellulose and BapA expression on biofilm and individual cell morphology at high resolution. To perform our studies we applied Atomic Force Microscopy (AFM) in combination with light microscopy. AFM is a powerful technique for imaging biological samples at the nanometer to micrometer scale under nondestructive conditions [24-29].

## Results

### Morphotypes on agar plates at high-resolution

**Figure 2:**
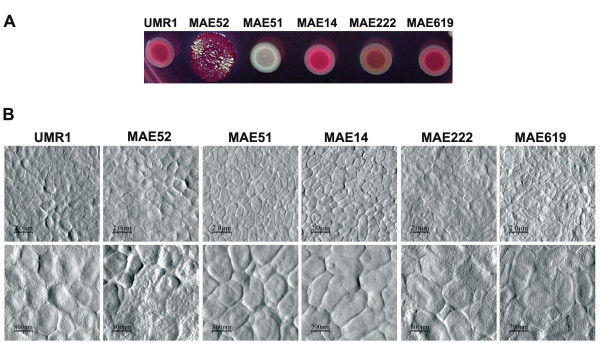
Colony morphology of *Salmonella *Typhimurium. The distinct morphotypes of UMR1 and its mutants MAE52 (*csgD*^++^), MAE51 (*csgD*), MAE14 (*csgBA*), MAE222 (*bcsA*) and MAE619 (*bapA*) are visualized on LB agar plates supplemented with congo red and coomassie blue (A) and on the high resolution AFM images (B).

**Figure 3:**
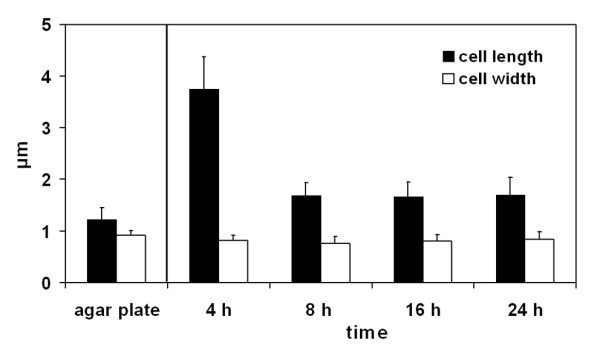
Cell dimensions of bacteria grown on agar plates and of bacteria within biofilms grown for 4, 8, 16 and 24 h in a liquid. Each value represents the average of measurements of 20 bacteria. Error bars represent standard deviations.

Curli and cellulose are the predominant matrix-compounds in *Salmonella enterica *serovar Typhimurium (*S*. Typhimurium) biofilms [23] (Fig. 1). The disruption of both or either of these components leads to distinct changes in colony morphology on Congo Red agar plates [23] (Fig. 2A). To analyse these changes at high resolution with AFM, colonies of *S*. Typhimurium UMR1 and its mutated derivatives were carefully transferred onto a cover slip and subsequently analysed by AFM in contact mode. Our data show that the colonies consisted of small tightly associated cells with a roundish cell shape (approx. 1.2 μm in diameter) (Fig. 2B and Fig. 3). Figure 2B shows that the surface of the wild type and in particular of the mutant MAE52, a strain overproducing curli and cellulose due to a point mutation in the promoter region of the *csgD *gene, was covered by a layer of extracellular material. This material was not visible on a mutant lacking the global regulator CsgD (MAE51) and a CsgBA mutant (MAE14), lacking expression of the major curli subunit CsgA and the surface-exposed CsgB nucleator (Fig. 2B). Subtle differences were observed between the wild type and a *bcsA *mutant (MAE222), disrupted in the gene encoding the bacterial cellulose synthase. Recently, Latasa *et al*. have demonstrated that besides curli and cellulose, CsgD also coordinates the production of BapA, a surface protein required for biofilm formation in *Salmonella enterica *serovar Enteritidis. We therefore tested the effect of a *bapA *mutation on colony and cell morphology of *Salmonella *Typhimurium (Fig. 2A and 2B). Our data show that the wild type and the mutant MAE619 were indistinguishable regarding colony morphology both on a Congo red agar plate as well as on our high-resolution AFM images. This result agrees with the earlier observation that a BapA deficient strain produced similar levels of cellulose and curli as the wild type [21].

### Imaging of *Salmonella *biofilms

**Figure 4:**
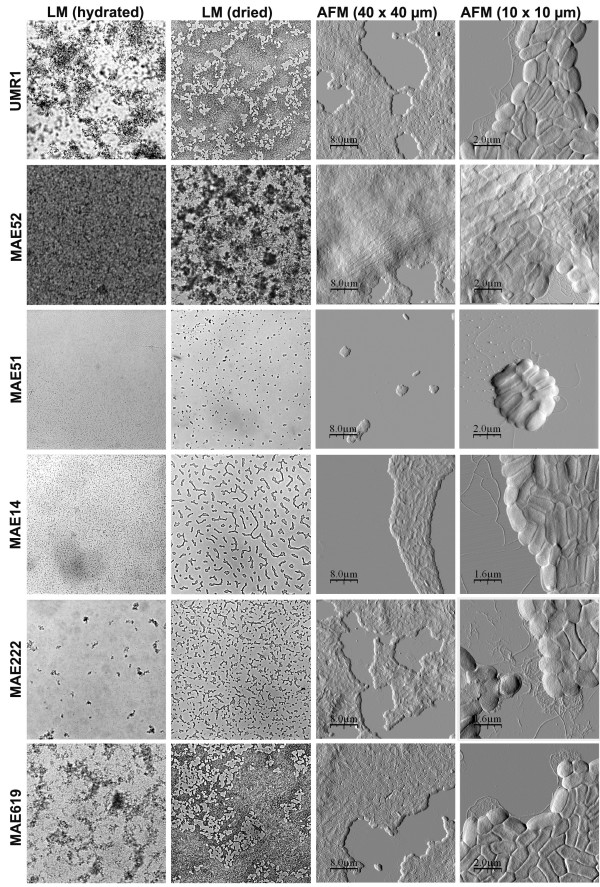
Cell and biofilm morphology of *Salmonella *Typhimurium UMR1 (wt) and its mutants MAE52 (*csgD*^++^), MAE51 (*csgD*), MAE14 (*csgBA*), MAE222 (*bcsA*) and MAE619 (*bapA*) after growth for 24 hours. The first column shows light microscope (LM) images (with a frame width of approx. 0.6 mm) before drying, the second column light microscope images at the same magnification after drying. The third and fourth columns show AFM images at two different scan sizes.

**Figure 5:**
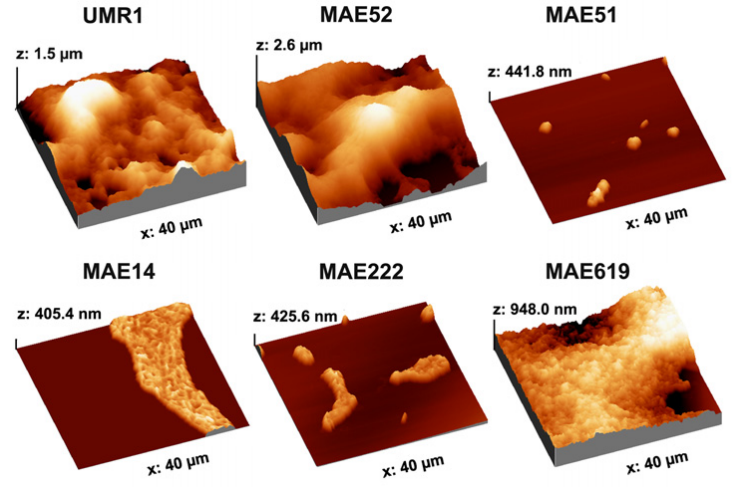
Topography AFM images of biofilms formed by UMR1 (wt), MAE52 (*csgD*^++^), MAE51 (*csgD*), MAE14 (*csgBA*), MAE222 (*bcsA*) and MAE619 (*bapA*) after growth for 24 hours on mica surfaces.

We wanted to analyse the different morphotypes also on biofilms grown on an abiotic surface in a liquid. We allowed the bacteria to form biofilms on the mineral surface mica, which was submerged in a rich growth medium. For immobilisation the samples were air-dried at room temperature prior to AFM and light microscopy analysis. To follow possible changes induced by the dewetting and drying processes, we also analysed the biofilms before drying in their hydrated state with the light microscope. Figure 4 shows that after 24 h the mica surface was entirely covered with biofilm by the wild type strain UMR1. The AFM data show that the biofilm consisted of tightly associated bacteria, similar to the colonies on the agar plate. We noticed however that the cells were longer (approx. 1.7 μm in length) and not as shrunken and roundish as the cells grown at the air-interface on agar (Fig. 2B and Fig. 3). At the edges of the biofilm flagella (approx. 20 nm in height) and some other thinner fimbrial structures (approx. 5 nm in height) could be detected. At some locations extracellular matrix was also seen on and between the cells in the biofilm, but to lower extents than in the colonies grown on agar. The topography data indicate that the biofilm consisted of multiple layers and that the thickness varied between different areas (Fig. 5).

### Impact of curli, cellulose and BapA on 24 h biofilms

With the aim to better understand how curli, cellulose and BapA contribute to biofilm formation on submerged slides, we compared the wild type biofilms with those formed by the curli, cellulose and BapA mutants. Figures 4 and 5 show that the curli and cellulose overproducing strain MAE52 formed a thicker and denser biofilm compared to the wild type. The AFM images showed more fimbrial structures at the edges of the cells than in the wild type (Fig. 4). Like with the bacteria grown on the agar plate, large amounts of extracellular material were detectable on and between the biofilm-associated cells. In contrast to MAE52, the *csgD *mutant MAE51 was deficient to form a biofilm (Fig. 4 and Fig. 5). Only individual cells and insular very small cell aggregates could be observed after 24 hours. The AFM images showed flagella, but no fimbriae or other apparent biofilm components. Also the *csgBA *mutant failed to form an area-wide biofilm within 24 h, instead, a layer of loosely attached individual bacteria was seen in the hydrated sample (Fig. 4). During the dewetting process these loosely attached cells were arranged into branched colonies on the surface. Similar, but more irregular colonies were observed on the dried biofilm samples of the cellulose mutant MAE222 (Fig. 4). However, light microscope images of the hydrated biofilm show that, in contrast to MAE14, this mutant partly retained the ability to form three dimensional loosely attached cell aggregates. This led us to suggest that curli are more important for the formation of the initial cell aggregates than cellulose. Like in the *csgD *mutant MAE51, we could not detect any of the thin fimbrial structures in the curli mutant MAE14, demonstrating these structures were made up of curli. On the other hand, wild type levels (or even slightly more) of the extracellular material could be observed in the cellulose mutant MAE222. Probably, this material mainly consists of curli. However, we cannot rule out that another so far uncharacterized polysaccharide, which has previously been suggested to exist [19], might be one of the compounds of the extracellular material.

Similar to the agar plates we were not able to detect a significant difference between a *bapA *mutant (MAE619) and the wild type in our liquid biofilm assay (Fig. 4 and Fig. 5). In contrast to the curli and cellulose mutant, the *bapA *deficient strain formed a dense biofilm within 24 hours and curli expression appeared to be the same as in the wild type.

### Monitoring biofilm expression over time

**Figure 6:**
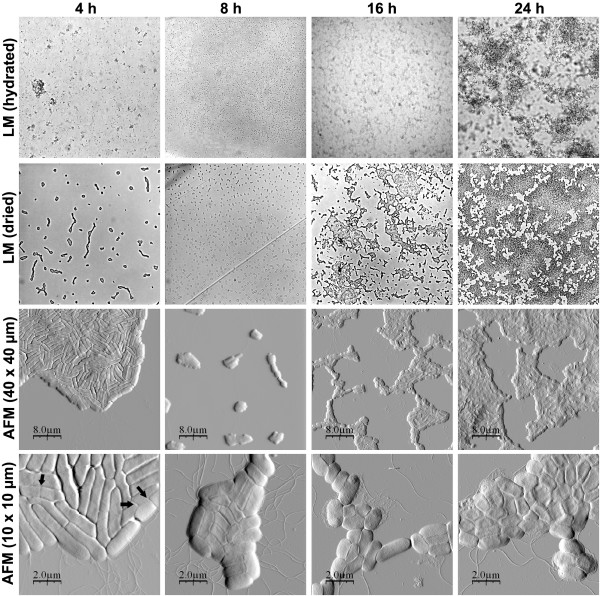
*Salmonella *Typhimurium UMR1 biofilm development after 4, 8, 16 and 24 h. The first row shows light microscope images (with a frame width of approx. 0.6 mm) before drying, the second row light microscope images at the same magnification after drying and the third and fourth rows show AFM images at two different scan sizes. The arrows highlight the appearance of division septa after 4 h.

The fact that we were able to monitor the expression of curli and flagella in *Salmonella *biofilms, prompted us to follow the expression of the extracellular structures during the growth of the biofilm. Biofilms of strain UMR1 were grown for 4, 8, 16 and 24 h and were analysed by microscopy as described above. After 4 h individual cells and only a few sporadically dispersed cell aggregates were observed on the mica surfaces covered with water (Fig. 6). The AFM images revealed that the bacteria were distinctly elongated (up to 4 μm in length) at this stage (Fig. 3 and Fig. 6). Many of them were in the process of dividing as the appearance of division septa indicated. Flagella were detectable in moderate numbers, but no other extracellular structures could be observed. After 8 h a layer of individual cells, which however did not form any aggregates, covered the surface. We assume that the cells were loosely attached to the surface because the dewetting process arranged the cells into a pattern of small periodically dispersed colonies. The AFM data show that the cell length of bacteria grown for 8 h was significantly decreased (approx. 1.7 μm) and no division septa were visible, indicating that the growth rate had decreased. Compared to the 4 hour time point flagellar expression was clearly increased. After 16 h the formation of three dimensional cell aggregates, firmly attached to the surface, had started. At this time point we could also begin to see large amounts of extracellular material that we earlier concluded had curli as the major constituent. After 24 h a confluent three dimensional biofilm was formed on the surface as described above.

### Biofilm formation in mutated strains over time

**Figure 7:**
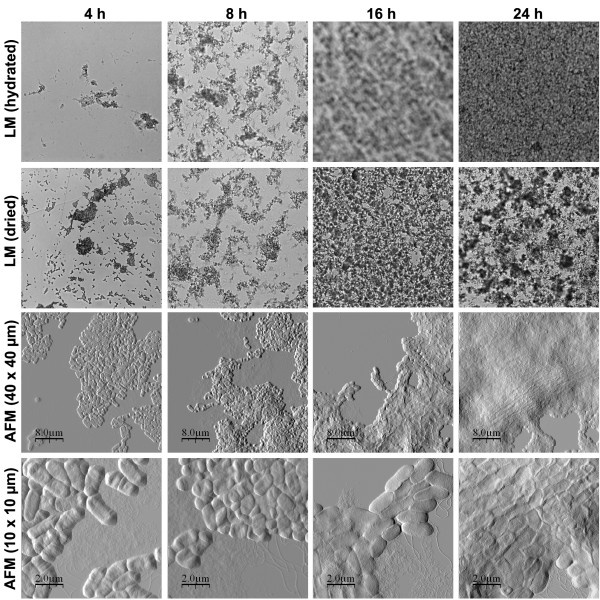
*Salmonella *Typhimurium MAE52 biofilm development after 4, 8, 16 and 24 h. The first row shows light microscope images before drying, the second row light microscope images after drying and the third and fourth rows show AFM images at two different scan sizes. The frame width of each image is approx. 0.6 mm.

**Figure 8:**
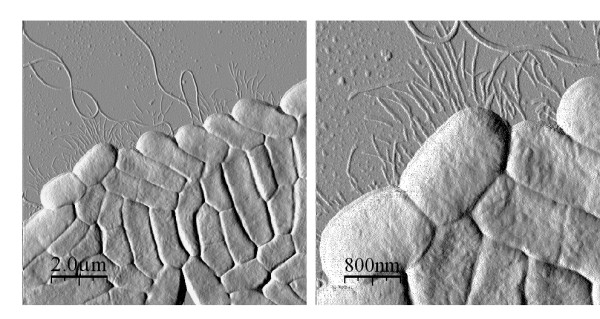
High resolution AFM images of pili-like fimbrial structures and flagella in the curli mutant MAE14 after 4 h at two different scan sizes.

To investigate the effect of overexpression of curli and cellulose on the time course of biofilm formation, we compared biofilms of MAE52 grown for 4, 8, 16 and 24 h to the wild type (Fig. 7). The light microscope images show that the cells started to form aggregates already within 4 h, apparently tightly bound to the surface as they were hardly influenced by the drying procedure. Growth of the aggregates rapidly gave rise to an area-wide biofilm on the surface. AFM analysis revealed that flagella and large amounts of biofilm matrix were produced at all time points (Fig. 7). Noticeably, MAE52 exhibited a more irregular surface structure with indentations than the wild type. We also analysed biofilm formation at the earlier time points for mutants MAE51, MAE14, MAE222 and MAE619 (summarized in Table 2). Interestingly, we were able to detect some other pili-like fimbriae in the curli deficient mutant MAE14 after 4 and 8 h (Fig. 8). Though less abundant these pili were also seen in the *bapA *mutant MAE619 and the cellulose mutant MAE222, but at no time point in the wild type. The height (6 nm) and the length (over 1 μm) of these features are in accordance with the properties of Type 1 pili, previously characterized by Korhonen *et al*. [30].

**Table 1:** Strains used in this study.

Bacterial Strain	Genotype	Reference or Source
*Salmonella *Typhimurium ATCC14028		

UMR1	ATCC14028-1s Nal^r^	[41]
MAE14	UMR1 Δ*csgBA101::km*^r^	[20]
MAE32	UMR1 P *csgD2*	[18]
MAE51	MAE32 Δ*csgD101*	[20]
MAE52	UMR1 P *csgD1*	[18]
MAE222	UMR1 *bcsA101*::MudJ	[44]
MAE619	UMR1 *bapA::cat*^r^	This study

**Table 2:** Flagella, curli and pili expression and aggregating behaviour of wild type strain UMR1 and its mutants grown on mica surface over time.

Strain		4 h	8 h	16 h	24 h
UMR1	Flagella	++	+++	+++	++
	Curli	-	-	+++	+++
	Pili	-	-	-	-
	Cell aggregation	+/-	-	++	+++
MAE52	Flagella	++	+++	+++	++
	Curli	++	++	+++	+++
	Pili	-	-	-	-
	Cell aggregation	++	+++	+++	+++
MAE51	Flagella	++	+++	+++	++
	Curli	-	-	-	-
	Pili	-	-	-	-
	Cell aggregation	-	-	-	-
MAE14	Flagella	++	+++	+++	++
	Curli	-	-	-	-
	Pili	++	+	+	-
	Cell aggregation	-	-	-	-
MAE222	Flagella	++	+++	+++	++
	Curli	+	+	+++	+++
	Pili	-	+	-	-
	Cell aggregation	-	-	+	+
MAE619	Flagella	++	++	+++	++
	Curli	-	-	++	+++
	Pili	+	+	-	-
	Cell aggregation	-	-	++	+++

- not detected

+ detected at low amounts

++ detected at moderate amounts

+++ detected at high amounts

## Discussion

In this study we have combined high-resolution atomic force microscopy with light microscopy to study the roles of curli, cellulose and BapA for the morphology of bacteria grown as colonies on agar plates and within a biofilm on a solid surface in liquid. Under both growth conditions bacteria were visible on the AFM images as tightly associated cellular units, resembling a tissue-like structure. This characteristic appearance was to a lesser extent visible on scanning electron micrographs and images of ultra-thin sections [20] as well as on confocal scanning laser microscopy [15] previously taken of plate grown cells, most probably due to differences in the way of sample preparation and imaging technology.

We noticed that the bacteria grown at the air-interface were rounder, smaller in size and more tightly attached to each other, whereas the biofilm-associated bacteria grown in liquid medium retained a more elongated structure. We were able to detect extracellular matrix, predominantly consisting of curli fibres, as a layer on and between the cells grown on agar. In the liquid biofilm assay curli fibers were visualised as thin fimbrial structures at the edges of the biofilms and on and between the bacteria by the AFM, resembling largely the electron micrographs of curli published by Chapman *et al*. [12].

Noticeably, no striking differences in morphology were observed between the curli mutant MAE14 and the CsgD mutant under either condition. Thus, in contrast to curli, cellulose expression could not be directly visualised by AFM. One explanation for this finding could be that the sensitivity of cellulose to the washing and drying procedure makes its visualisation difficult [31,32]. Another possibility is that cellulose is expressed at relatively low levels and that it is not secreted into the environment. Cellulose might be embedded in the LPS layer and thereby alter the ability to adhere to the surface and to other cells. Our light microscopy data from the biofilm assay show that both curli and cellulose are necessary for the formation of a biofilm within 24 h, wherein curli seem to be more important for the formation of the early cell aggregates than cellulose.

The results from the time course of biofilm growth show that the wild type began to form aggregates and to bind firmly to the surface first after curli production had started between 8 and 16 h. Thus, curli seem to be indispensable for the formation of cell aggregates and for making strong attachment to a surface.

BapA has recently been discovered as a large secreted protein in *Salmonella *Enteritidis, which is loosely associated with the cell surface and required for pellicle formation at the air-liquid interface [21]. Noticeably, the results from our biofilm assays show that neither the biofilm and colony morphology nor the ability to form a biofilm within 24 h on a submerged surface was altered in a *bapA *mutant in *S*. Typhimurium. Since BapA is a large surface protein one would expect that the disruption of BapA would cause changes in the cell surface appearance. Similar to the wild type the surface of this mutant was characterized by an orange-peel appearance that already has been discussed in earlier studies [26,33].

An interesting finding was that in some mutants, but not in the wild type, pili-like structures occurred at the early time points of biofilm formation. These pili-fimbriae were most abundantly found in the curli mutant MAE14 and to a lesser extent in the *bapA *and the cellulose mutants. This finding leads us to suggest that expression of pili, curli and other surface structures might be coregulated.

Several immobilisation strategies have been described in the literature for studying bacteria with AFM, including chemical fixation, air-drying, several kinds of coating or the use of porous membranes [29,34-37]. However, most of them interfere with the natural ability of bacteria to attach to a surface and are therefore not suitable for the study of biofilms. In our study the AFM operations were performed on plate grown colonies and on biofilms. Since colonies are well attached to the agar plate and grow at the air-interface, the colonies could be directly imaged with the AFM, ensuring a simple and non-destructive way of sample preparation, which to our knowledge has not been reported elsewhere. For imaging of the biofilms, grown in a liquid, we air-dried the samples prior to AFM analysis. AFM imaging of dried bacterial samples is well established [26,38-40], leading to the strong attachment necessary for the AFM operation. Moreover, AFM on dried bacteria has been demonstrated to allow for high-resolution imaging of flexible and moving extracellular structures such as flagella, fimbriae and biofilm matrix components [26], which would be difficult to detect in liquid. According to previous studies drying does not seem to distort or destroy the bacterial morphology [26]. Being aware that the drying process could induce changes in the overall biofilm structure, we analysed the biofilms before and after drying with the light microscope. We noticed that drying caused the formation of small colonies on the surface, when bacteria were loosely attached to the surface, e.g. in some mutants or at the early time points (Fig. 4 and Fig. 6). This should however not affect the parameters we have studied, such as curli, flagella and pili expression as well as cell size and shape.

Our work demonstrates the power of AFM as a useful tool to monitor the expression of genes involved in bacterial morphology during biofilm formation. The power of the methodology would become even more significant, once a way has been found to image biofilms in their hydrated state under water with the AFM. However, there are several challenges that have to be overcome, such as strong interactions between the cells and the scanning tip as well as difficulties in the immobilisation of the hydrated biofilms [29].

## Conclusion

Our work deepens the knowledge about the role of curli, cellulose and BapA in biofilm formation. Our data illustrates that curli and cellulose but not BapA have a major impact on the formation and the morphology of a biofilm, wherein curli seem to be more important for the formation of cell aggregates than cellulose. Here, we demonstrate that AFM is a useful tool for studying bacterial biofilms and colony morphology at high resolution, complementing recent approaches, such as Congo Red plates, pellicle and biofilm microtiter plate assays or the flow chamber system.

## Methods

### Bacterial strains

Bacterial strains employed in this study are listed in Table 1. *Salmonella *enterica serovar Typhimurium UMR1 was chosen as it expresses the rdar morphotype, characterized by the expression of cellulose and curli fimbriae [41,42]. Strain MAE619 (*bapA*::cat^r^) was constructed according to the Datsenko method [43]. Briefly, the chloramphenicol cassette from plasmid pKD3 was amplified using the primers bapA_start 5'-ATGCGTCTACTCGCCGTGGTTTCGAAATTGACTGGCGTCTGTG TAGGCTGGAGCTGCTTC-3' and bapA_stop 5'-TCAACCGCTGGTTATCAGG TGGTTGTTCTGCAACAGATCACATATGAATATCCTCCTTAGT-3'. The purified PCR product was subsequently electroporated into UMR1 containing pKD46 (amp^r^). Mutants were selected for the gain of chloramphenicol resistance and the loss of ampicillin resistance and were verified by PCR using the control primers STM2689before 5'-AATCCATCAGGAGCTGATTT-3' and STM2689after 5'-ATAGTTTATCCCTTTTAAATATCACCG-3'.

### Growth conditions

*Salmonella *strains were routinely grown in Luria broth medium (10 g Bacto tryptone, 5 g Bacto yeast extract, 10 g NaCl per liter) at 37°C overnight. *Colony formation: *For analysis of Congo Red binding 3 μl of an overnight culture suspended in the same volume of water were spotted onto LB without NaCl agar plates supplemented with Congo Red (40 μg ml^-1^) and Coomassie brilliant blue (20 μg ml^-1^). Plates were incubated at 28°C for 24 hours. The development of the colony morphology and dye binding was analysed over time. For the AFM analysis bacteria were spread on LB plates without NaCl and without the binding dyes and incubated for 24 h at 28°C. *Biofilm formation: *Mica slides Grade V-4 (SPI^® ^Supplies, USA) were cleaved in thin layers (approximately 1 cm^2^), and subsequently transferred into 6 well Corning culture plates (4 cm diameter), each well containing 3 ml tryptonic broth medium (10 g tryptone per liter). The mineral surface mica was used as a substratum as it is atomically flat, giving a distinctly lower background than glass in the AFM analysis. Overnight cultures were diluted 1:100 into the 6 well plates, which were then incubated for 4 h, 8 h, 16 h or 24 h at 28°C without shaking.

### Light microscopy

After incubation the biofilm-covered mica slides were transferred into new petri dishes filled with double distilled water and analysed in their hydrated state with an Eclipse E400 light microscope (Nikon instruments) and images were taken with a connected CCD camera (Nikon instruments). Light microscopy was performed a second time after air-drying on the dried biofilms. All light microscopy images were taken at the same magnification. The frame width of each image corresponds to approx. 0.6 mm.

### AFM sample preparation

*Colonies grown on agar: *Due to the rough and dry colony appearance it was possible to carefully peel off the colonies of strains UMR1, MAE52 and MAE619 from the agar plates, to transfer them onto glass cover slips and to image them subsequently with the AFM. The smoother colonies of MAE51, MAE14 and MAE222 were carefully lifted up together with some of the underlying agar, transferred onto the cover slips and then analysed by AFM. *Biofilms: *After incubation the biofilm covered mica slides were dipped 3 to 4 times into double distilled water, air-dried at room temperature in a dust-free environment overnight and glued onto glass microscope slides using nail polish prior to the AFM analysis. Air-drying was chosen as an immobilisation strategy making the bacterial cells resistant to the lateral forces exerted by the scanning tip, without adding agents that interfere with the natural ability to form biofilms on surfaces.

### AFM operation

Bacteria were imaged with the Nanoscope Dimension 3100 and the BioScope SZ (Veeco Instruments Inc, NY, USA). The instruments were operated using the contact mode, an AFM operation mode where the force between the tip and the surface is kept constant during scanning by maintaining a constant deflection [24]. Images were obtained using V-shaped silicon nitride nanoprobe cantilevers MLCT-AUHW (Veeco) with a spring constant of 0.05 N/m. The data were analysed with the scanning probe software WSxM (Nanotec). Deflection images were used for illustration and topography images for measurements on cell and colony properties. The cell length and cell width of the bacteria was measured on height profiles acquired with the WSxM software. Each presented value represents the mean of measurements on 20 different bacteria. The level of flagella, curli and pili production as well as the ability to form cell aggregates was estimated by visual observations.

## Authors' contributions

KJ performed most of the laboratory work with the initial assistance of HT. AK constructed the MAE619 mutant. LB supervised the AFM work. KJ, SN, UR, LB and ÖM designed the study and analysed the data. KJ and ÖM wrote the manuscript. ÖM coordinated the study. All authors read and approved the final version of the manuscript.
